# Crepuscular Flight Activity of an Invasive Insect Governed by Interacting Abiotic Factors

**DOI:** 10.1371/journal.pone.0105945

**Published:** 2014-08-26

**Authors:** Yigen Chen, Steven J. Seybold

**Affiliations:** 1 Department of Entomology and Nematology, University of California Davis, Davis, California, United States of America; 2 USDA Forest Service Pacific Southwest Research Station, Chemical Ecology of Forest Insects, Davis, California, United States of America; United States Department of Agriculture, Beltsville Agricultural Research Center, United States of America

## Abstract

Seasonal and diurnal flight patterns of the invasive walnut twig beetle, *Pityophthorus juglandis*, were assessed between 2011 and 2014 in northern California, USA in the context of the effects of ambient temperature, light intensity, wind speed, and barometric pressure. *Pityophthorus juglandis* generally initiated flight in late January and continued until late November. This seasonal flight could be divided approximately into three phases (emergence: January–March; primary flight: May–July; and secondary flight: September–October). The seasonal flight response to the male-produced aggregation pheromone was consistently female-biased (mean of 58.9% females). Diurnal flight followed a bimodal pattern with a minor peak in mid-morning and a major peak at dusk (76.4% caught between 1800 and 2200 h). The primarily crepuscular flight activity had a Gaussian relationship with ambient temperature and barometric pressure but a negative exponential relationship with increasing light intensity and wind speed. A model selection procedure indicated that the four abiotic factors collectively and interactively governed *P. juglandis* diurnal flight. For both sexes, flight peaked under the following second-order interactions among the factors when: 1) temperature between was 25 and 30°C and light intensity was less than 2000 lux; 2) temperature was between 25 and 35°C and barometric pressure was between 752 and 762 mba (and declined otherwise); 3) barometric pressure was between 755 and 761 mba and light intensity was less than 2000 lux (and declined otherwise); and 4) temperature was ca. 30°C and wind speed was ca. 2 km/h. Thus, crepuscular flight activity of this insect can be best explained by the coincidence of moderately high temperature, low light intensity, moderate wind speed, and low to moderate barometric pressure. The new knowledge provides physical and temporal guidelines for the application of semiochemical-based control techniques as part of an IPM program for this invasive pest.

## Introduction

The invasive walnut twig beetle, *Pityophthorus juglandis* Blackman (Coleoptera: Scolytidae) [1], [2], is native to the southwestern USA and Mexico [3], but it has expanded its range in the USA to include nine western and five eastern states [4], [5]. In 2012 and 2013, it was detected in one county each in Ohio and Maryland, respectively, and is suspected to have invaded North Carolina from neighboring Tennessee [5]. In symbiosis with the newly discovered phytopathogenic fungus, *Geosmithia morbida* M. Kolařík, E. Freeland, C. Utley, & N. Tisserat sp. nov. (Ascomycota: Hypocreales) [6] [known collectively as thousand cankers disease (TCD)], the phloeophagous *P. juglandis* has driven an expanding pattern of mortality of black walnut trees (*Juglans* spp.) in the western USA [5], [7]. Underscoring the potential international significance of TCD to the world’s walnut culture, *P. juglandis* and *G. morbida* were reported in 2013 in northern Italy, also in association with black walnut trees grown in plantations for timber production [8].

The relatively recent range expansion of *P. juglandis* can be attributed to introductions *via* human-assisted movement of wood products leading to the establishment of satellite populations in areas with suitable hosts (typically eastern black walnut, *Juglans nigra* L.) [9]. Invasive satellite populations expand subsequently by natural flight dispersal or by local human-assisted movement (e.g., the population in Tennessee is centered on Knox Co., but has expanded to include nearly 20 counties in the state, P. L. Dallara et al. unpublished data). More recently discovered introductions in Maryland, Ohio, and Pennsylvania appear to have not expanded yet beyond the original counties where *P. juglandis* was introduced (P. L. Dallara et al. unpublished data).

Little is known about the physical factors that dictate the flight behavior of *P. juglandis* or of any of its congeners. However, numerous studies have been conducted on the interaction of selected abiotic factors with the flight behavior of other bark and ambrosia beetles (Scolytidae) [10]–[13]. Many of these studies have used bark or ambrosia beetle aggregation pheromones as a useful tool for luring this group of insects in flight studies. These studies have revealed distinctive seasonal flight patterns that may be species-specific. For instance, adult *Pseudohylesinus nebulosus* (LeConte) (Coleoptera: Scolytidae) initiates flight in March and flight activity peaks from March to May [10]. Flight continues into September. In contrast, the flight of *Scolytus unispinosus* (LeConte) does not start until late May or early June, and flight stops in late July or early August [10]. All species of Scolytidae in these studies were found to fly either in daylight and/or during crepuscular hours, some showing unimodal [14]–[16] and others showing bimodal [11], [12], [17] flight patterns. Temperature, followed by light intensity in some cases, are generally thought to be the primary abiotic factors affecting beetle flight activity [10], [18]–[21].

The discovery of a male-produced aggregation pheromone for *P. juglandis* [22] facilitated a preliminary assessment of the diurnal flight pattern of *P. juglandis* in northern California that revealed that the daily flight pattern switched during different seasons of the year: unimodal during late June/early July and bimodal from late August to early November [23]. Regardless of the season, approximately 50% of the beetles were caught at or near the dusk period. The relationship between *P*. *juglandis* flight and temperature was Gaussian, with the peak activity at 23 to 24°C. Flight activity gradually declined as temperature increased or decreased, and activity ceased when temperature was below 15°C or above ca. 34°C. The study led to the hypothesis that low or declining light intensity (and perhaps other abiotic factors) may interact with temperature to elicit maximum crepuscular flight responses from a population of adult *P. juglandis* [23].

To test the hypothesis of interacting abiotic factors and to validate the summer/fall diurnal patterns at an earlier point in the season (i.e., May–June) and for a longer duration, we investigated the diurnal flight pattern from 8 May to 17 September, 2012 and measured concomitantly the temperature, light intensity, wind speed, and barometric pressure. We hypothesized that *P. juglandis* exhibited a shifting modality of diurnal flight with advancing season (i.e., bimodal–unimodal–bimodal). We also hypothesized that the four abiotic variables affected *P. juglandis* activity differently and interacted to govern the crepuscular flight activity of *P. juglandis*. Weekly trap catches from 29 August, 2011 to 2 June, 2014 (i.e., during the previous and beyond the current diurnal studies) were also compiled and reported here to document the seasonal flight pattern of *P. juglandis*. We anticipated that the flight data from the diurnal and seasonal surveys would be valuable as guidelines for conducting future pest management activities with *P. juglandis*.

## Materials and Methods

### Ethics statement

No specific permissions were required for locations/activities involved in the study. The field studies did not involve endangered or protected species.

### Study site

The study site was a native riparian forest stand of northern California black walnut, *Juglans hindsii* (Jeps.) Jeps. *ex* R. E. Sm., Fremont’s cottonwood, *Populus fremontii* S. Wats., and valley oak, *Quercus lobata* Née, located along the north fork of Putah Creek in Davis (38°32′20.66″ N, 121°44′21.42″ W, approx. 16 m elev.) in Yolo Co., California, USA.

### Flight trapping and beetle handling

Five, twelve-unit black plastic multiple funnel traps (Contech Enterprises Inc., Delta, B.C., Canada) were baited with the *P. juglandis* aggregation pheromone [22], [24] and spaced at a distance greater than 50 m from each other at the study site described above. Traps were placed 3 to 5 m from the main stem of a *J. hindsii* tree and on top of a 3 m pole [24]. The pheromone was formulated (neat) for release by filling and capping a 15 ml polyethylene bottle (Contech product #100000582/583), which was hung with a wire from the sixth funnel in the trap. A collection cup with ∼100 ml of ethanol-free, propylene glycol-based antifreeze was attached to each trap [24]. Traps were first emptied weekly at 0800 h of every Monday starting 7 November, 2011 to 7 May, 2012. Traps were then emptied at nine time points (0600, 0800, 1000, 1200, 1400, 1600, 1800, 2000, and 2200 h; all times were Pacific daylight time) daily from 8 May to 17 September, 2012, to assess daily flight behavior. Diurnal trap catches (Appendix S1) recovered at all the time points contained *P. juglandis* trapped during a two-hour period except that of 0600 h, which included *P. juglandis* caught during an eight-hour period (i.e, from 2200 h the previous day to 0600 h the current day). Weekly emptying of traps resumed on 18 September, 2012 and continued until 2 June, 2014. The sexes of trapped specimens of *P*. *juglandis* were separated and identified under a Zeiss Stemi 2000 dissecting stereomicroscope (6.5x–40x magnification) according to morphological characters described in [24], [25]. Weekly *P. juglandis* catches (Appendix S1) from 29 August to 7 November, 2011 from the previously published data set [23] and from 8 May to 17 September, 2012, were calculated from the bihourly catches collected over those periods. Sex ratios (i.e., percentage of males) from weekly *P. juglandis* catches were calculated from trap catch data from weeks when the total catch exceeded 50.

In this note, we have elected to use the original family-level nomenclature for bark and ambrosia beetles (Coleoptera: Scolytidae) based on the argument presented in [1] and a more extensive treatment of the issue developed by D. E. Bright (personal communication) and published in his third supplement to the world catalog of the Scolytidae and Platypodidae [2]. In essence, morphological and fossil evidence of adult scolytids support the family-level treatment, whereas similarity in scolytid and curculionid larval morphology supports a subfamily placement. Because this issue is not entirely resolved, we prefer to take the more conservative approach of using the original nomenclature.

### Measurement of abiotic factors

Ambient temperature, wind speed, and barometric air pressure data (Appendix S1) were recorded with a Vantage Pro2 Weather Station (Davis Instruments Corp., Hayward, CA, USA) placed ca. 10 m to the east of one of the *J. hindsii* trees and one of the survey traps. Light intensity was obtained by using a Visible Light SD Card Datalogger (Sper Scientific, Scottsdale, AZ, USA) mounted on a 3-m metal pole placed ca. 8 m west of the weather station. Data were recorded at 10-min intervals. The mean temperature, wind speed, barometric pressure, and light intensity within each bihourly time period were calculated and used to examine their effects on *P. juglandis* flight activity.

### Statistical analysis

All statistical analyses were conducted in SAS v. 9.2 [26]. A critical level of *α* = 0.05 was used for all analyses. The number of wk on which more male *P. juglandis* was trapped was compared to the number of wk on which more female *P. juglandis* was trapped by using Pearson’s chi-square test. During the diurnal flight study (8 May, 2012 to 17 September, 2012), there were a total of 1205 *P. juglandis* trap catches, 855 temperature data points, 856 light intensity data points, 873 barometric pressure data points, and 1205 wind data points. The number of data points was unequal because of various equipment malfunctions during the study. Since *P. juglandis* trap catches (number of *P. juglandis* per trap per interval) were not normally distributed, they were analyzed with a non-parametric two-way ANOVA (PROC GENMOD in SAS) with time interval (nine levels) and sex (two levels) of *P. juglandis* as two factors and with dates as repeated measurements. The *P. juglandis* catches conditional on a normally distributed error were modeled as a Poisson distribution, and *P. juglandis* catches were linked to their expected values with a logarithm function [26].

The measurements of the abiotic factors were analyzed to understand the variation in their diurnal patterns. Ambient temperature, light intensity, and wind speed from the nine time points within a day were not normally distributed and were analyzed separately by non-parametric Kruskal-Wallis tests with multiple mean comparisons following methods in [27]. Barometric pressure data were analyzed by one-way ANOVA (PROC GLM in SAS) since they met the model assumptions of normality (by the Kolmogorov-Smirnov *D* statistic) and variance homogeneity (by Levene’s test).

To delineate abiotic factors that might affect *P. juglandis* flight behavior, ambient temperature, light intensity, wind speed, and barometric pressure data were each regressed against *P. juglandis* catches (separately by sex) (SigmaPlot 12.0; Systat Software, Inc., San Jose, CA, USA). Data were checked for outliers and those with Studentized residuals greater than eight were excluded from the regression. Following the method of [28], a regressed variable (e.g., temperature) was divided into 10–20 classes and the greatest value of the other (or dependent) variable (i.e., *P. juglandis* catch) in each class was used for regression. Class width was determined by first subtracting the lowest from highest value for each of the abiotic variables. This difference was then divided by integers between 10 and 20 to yield an integer number of class widths. Temperature data were divided into 16 classes with an increment of 2°C. Light intensity data were divided into 18 classes with an increment of 200 lux. Wind data were divided into 15 classes with an increment of 0.8 km/h. Barometric pressure data were divided into 14 classes with an increment of one mbar. If the number of data points in a certain class was fewer than four points, then these points were grouped into the next higher class. The Gaussian equation, *Y* = *α*×*exp*(−(*X*−*X0*)^2^/2*b*), was used to fit the relation between *P. juglandis* catches (*Y*) and temperature or barometric pressure (*X*). The exponential decay equation, *Y* = *α*×*exp*(−*bX*), was used to fit the relation between *P. juglandis* catches (*Y*) and light intensity or wind speed (*X*).

We evaluated various models to examine the combined effects of ambient temperature, light intensity, wind speed, and barometric pressure and their interactions on *P. juglandis* flight (PROC GENMOD). The *P. juglandis* catches conditional on a normally distributed error were modeled as a Poisson distribution. The expected values of *P. juglandis* catches and the linear predictor were linked with a logarithm function. A total of 94 models were evaluated (Appendix S2). Terms included in the models were first-order terms (ambient temperature, light intensity, wind speed, and barometric pressure; all four appeared in 87 of 94 models); second-order interactions (each interaction appeared in 48 models); third-order interactions (each interaction appeared in nine models); and fourth-order interactions (appeared in one model). The first-order terms were uncorrelated with each other [all Pearson’s correlation coefficients less than 0.5 and variance inflation factors (VIFs) less than 2]. The Quasi-likelihood adjusted Akaike’s Information Criteria (*QAIC*), Δ*_i_*, *w_i_*, and evidence ratios (*ER*) were computed according to [29]. The model with the minimum *QAIC* is considered to be the best model. *Δ_i_* (* = *QAIC*_i_*–*QAIC_min_*) is the plausibility that the fitted model is the best model given the data (the larger the *Δ_i_*, the less plausible); *w_i_* indicates the relative likelihood of the model *i* being the best model given the data. Models with a *w_i_* greater than 0.1 were presented [30]; *ER* is the weight of the best model divided by the weight of the fitted model *j* (the closer the *ER* is to 1 the better the fit of the model).

## Results

### Seasonal pattern of *P. juglandis* flight

**Figure 1:**
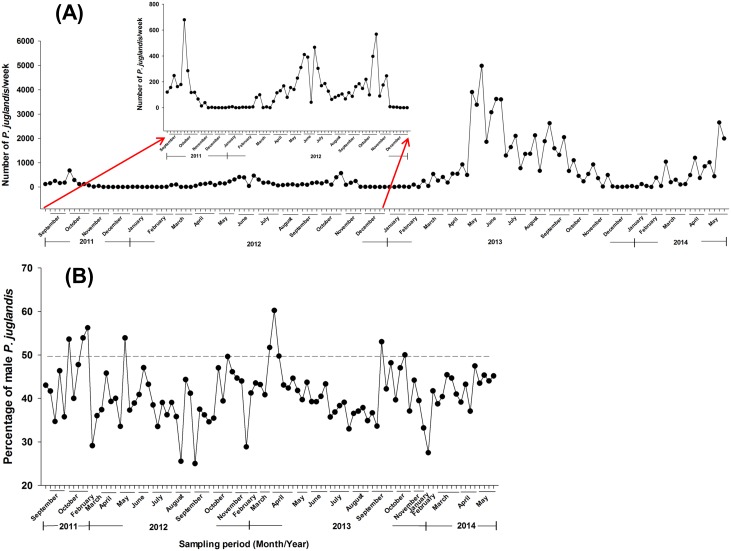
Weekly *Pityophthorus juglandis* total trap catches (A) and percentage of males in selected catches (B) from 29 August, 2011 to 2 June, 2014 in five Lindgren funnel traps. Hash marks along the x-axis denote the first day of each week. Percentages of males are presented for weeks when more than 50 *P. juglandis* were trapped. Inset in (A): rescaling of weekly *P. juglandis* catches from 29 August, 2011 to 31 December, 2012 to facilitate comparison of seasonal flight pattern with 2013 and 2014 when flight responses were higher.

A total of 73,842 *P. juglandis* were trapped between 29 August, 2011 and 2 June, 2014 (Fig. 1A), and 58.9% of the trap catch was female (Fig. 1B). Female flight response generally exceeded male flight response throughout most of the season as might be expected for a population response to a male-produced aggregation pheromone (Fig. 1B). Of the 97 wk, female catch exceeded male catch 89 times, which was significantly more than the instances (8) when male catch exceeded female catch (Fig. 1A; *X* ^2^ = 67.64, *P* = 0.00). There was no seasonal pattern to the occurrence of the male-biased responses. *Pityophthorus juglandis* flew at very low levels in December and January but generally initiated seasonal flight between late January and late February and ceased flight at the end of November (Fig. 1A). The preponderance of flight occurred between early May and late October, which bracketed the timing of our diurnal flight study. Thus, with *P. juglandis*, a sporadic spring “emergence” flight appears to take place from late January through March, followed by a more massive primary flight between May and July and a secondary flight in September and October.

### Diurnal patterns of *P. juglandis* flight and abiotic variables

**Figure 2:**
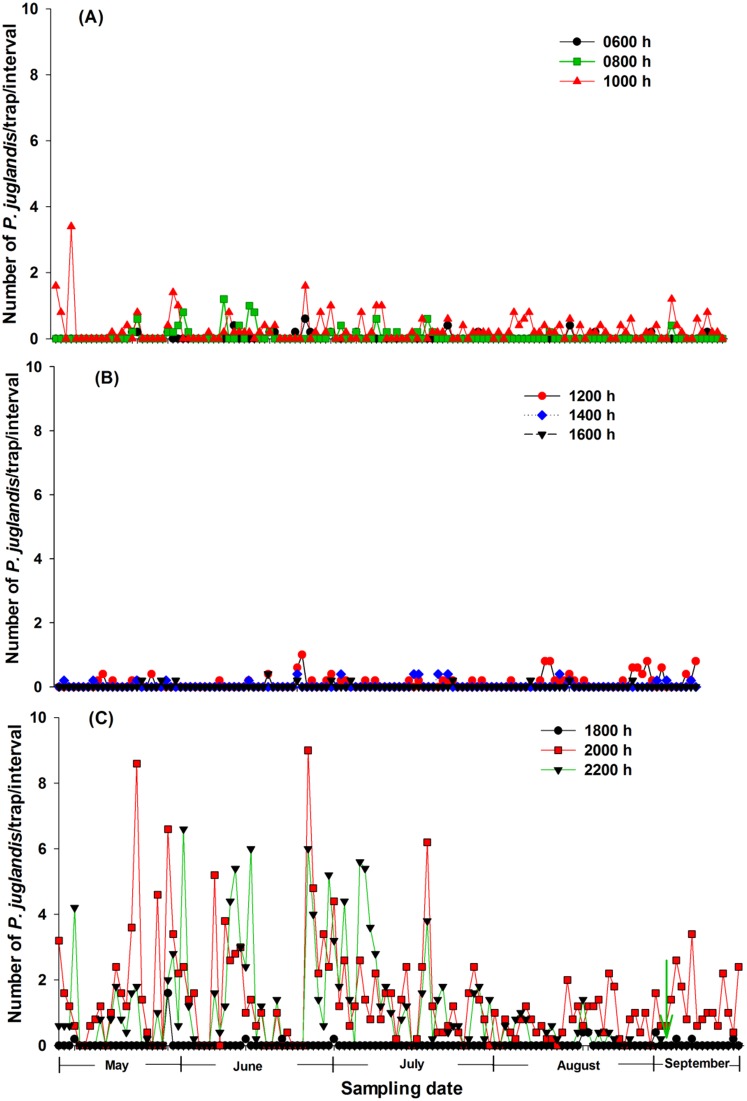
Daily *Pityophthorus juglandis* catches from 8 May to 17 September, 2012. (A) 0600–1000 h; (B) 1200–1600 h; (C) 1800–2200 h. Time intervals: 0600 h (2200 h of the previous day–0600 h the current day); 0800 h (0600–0800 h); 1000 h (0800–1000 h); 1200 h (1000–1200 h); 1400 h (1200–1400 h); 1600 h (1400–1600 h); 1800 (1600–1800 h); 2000 h (1800–2000 h); and 2200 h (2000–2200 h). Green arrow points to 3 September, 2012 when *P. juglandis* flight activity stopped between 2000 and 2200 h for the season. *N* = 133 days.

A total of 3,565 *P. juglandis* were trapped between 8 May and 17 September, 2012 (60.5% of the trap catch was female). Of the 134 days studied, no *P. juglandis* were caught during ten of the days (three days in May, five days in June, and two days in August). During 13 of the days (six days in May, three days in June, one day in July, and three days in August), no *P. juglandis* were trapped in the morning hours (between 0600 and 1200 h), whereas at least one *P. juglandis* was trapped in the afternoon/evening hours (between 1200 and 2200 h). During three of the days (one day each in May, June, and July), one *P. juglandis* was caught in the morning hours, whereas no *P. juglandis* were caught in the afternoon/evening hours. During the remaining 108 days, at least one *P. juglandis* was caught both in the morning and afternoon/evening hours. Thus, the number of days when no *P. juglandis* were caught either in the morning or in the afternoon/evening hours was 16 (i.e., unimodal flight activity), which is significantly fewer than the number of days (i.e., 108) that *P. juglandis* was caught both in the morning and afternoon hours (*X* ^2^ = 68.26, *P* = 0.00) (i.e., bimodal flight activity). Analysis of each 2-hr time interval also showed that *P. juglandis* was caught in the morning hours during most of the days (Fig. 2A). However, the most *P. juglandis* (largest peak) were caught between 1800 and 2200 h (Fig. 2C), and the least were trapped in the early afternoon (Fig. 2B). Neither *P. juglandis* catch in the morning nor during the entire day was well correlated with temperatures at 0600 of the day (Catch in the morning: *F* = 7.24, *P*<0.01, adj. *R* ^2^ = 0.062; Catch entire day: *F* = 0.47, *P*>0.05, adj. *R* ^2^ = −0.006). *Pityophthorus juglandis* flight activity declined between 2000 and 2200 h as the period of seasonal daylight shortened and activity during this 2-hr window ceased on 3 September, 2012 (Fig. 2C, green arrow). Light intensity at 2000 h was measured at zero lux beginning on 20 August, 2012.

**Figure 3:**
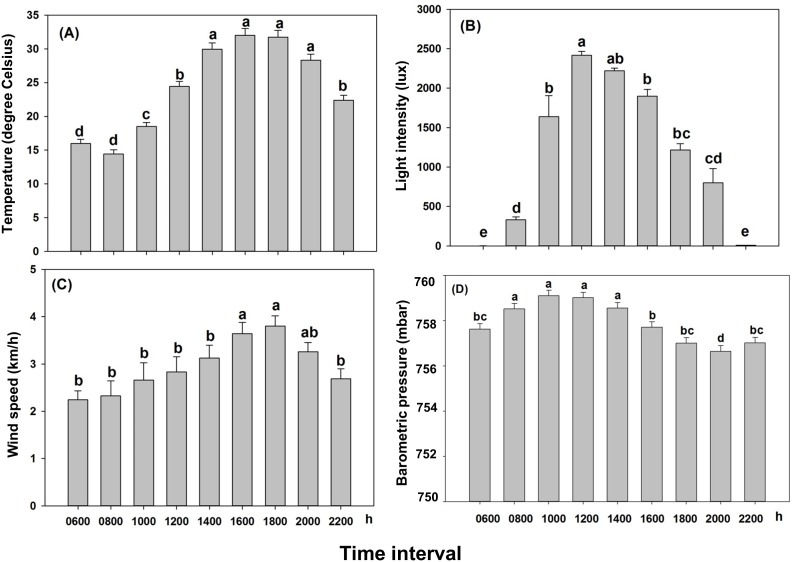
Environmental abiotic variables (mean + SE) at various time intervals of a day during the diurnal flight study of *Pityophthorus juglandis*. (A) Temperature; (B) Light intensity (B); (C) Wind speed; and (D) Air pressure. Time intervals: 0600 h (2200 h of the previous day–0600 h the current day); 0800 h (0600–0800 h); 1000 h (0800–1000 h); 1200 h (1000–1200 h); 1400 h (1200–1400 h); 1600 h (1400–1600 h); 1800 (1600–1800 h); 2000 h (1800–2000 h); and 2200 h (2000–2200 h). Different lower-case letters above bars denote significant difference between time points with each variable (*α* = 0.05). *N* = 134 days except for the time interval 0800 where *N* = 133 days.

**Figure 4:**
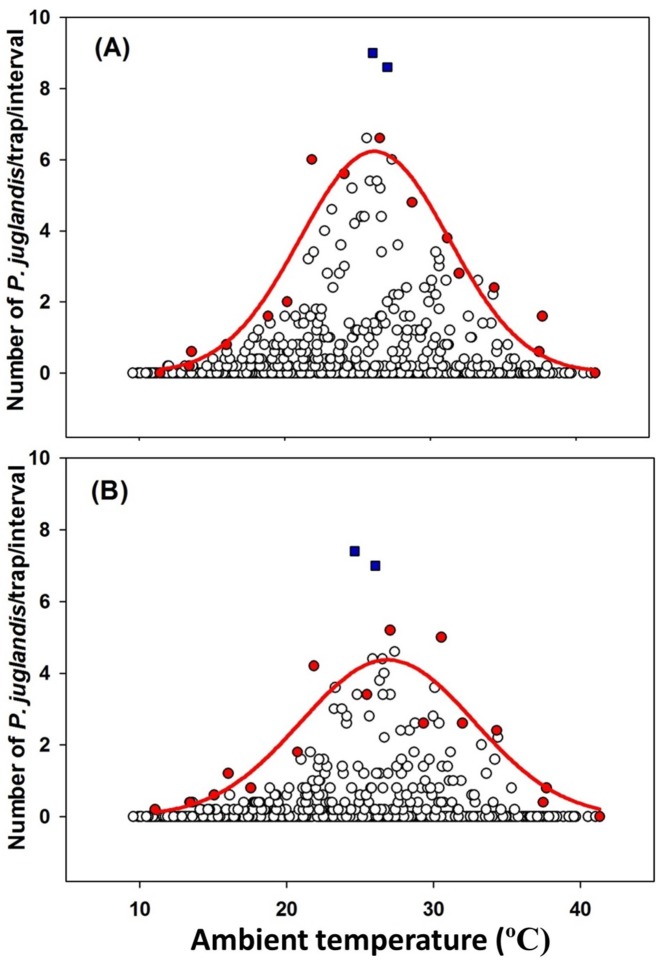
The Gaussian relationship (*Y* = *α*×*exp*(−(*X*−*X0*)^2^/2*b*)) between *Pityophthorus juglandis* catches and temperature (°C). (A) Female: *N* = 855, *F* = 63.38, *P*<0.001, *α* = 6.23, *b* = 5.11, *X*_0_ = 26.16, adj. *R* ^2^ = 0.89; (B) Male: *N* = 855, *F* = 28.87, *P*<0.001, *α* = 4.37, *b* = 5.89, *X*_0_ = 26.87, adj. *R* ^2^ = 0.79. Blue □: Outliers; Red ◯: Data points used to fit curves. These points were the greatest values of trap catches from each of the temperature classes [28].

**Figure 5:**
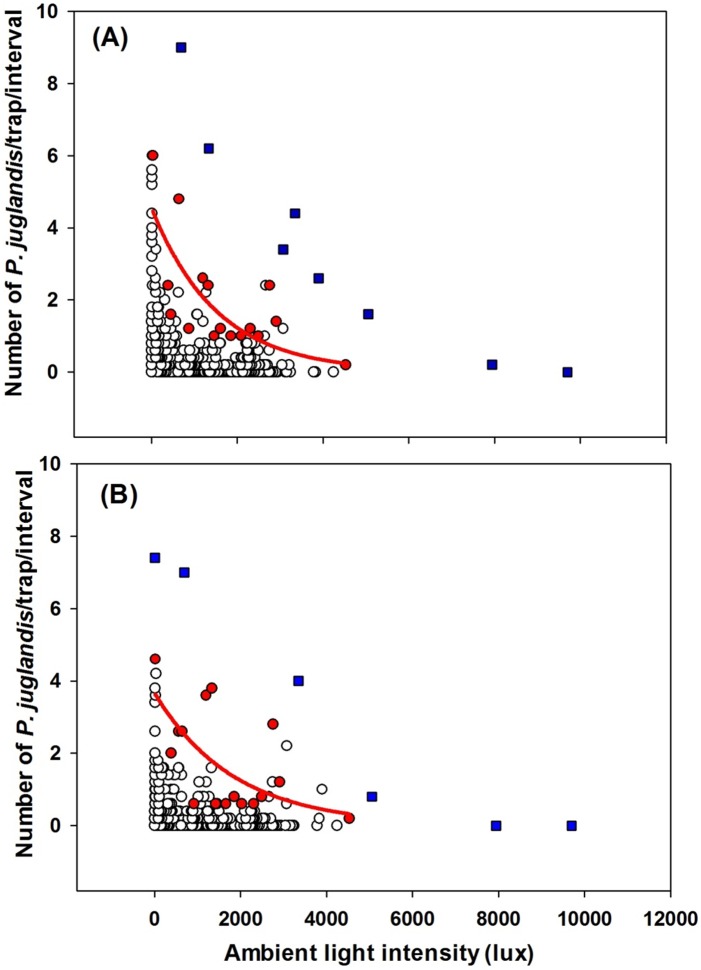
The exponential decay relationship (*Y* = *α*×*exp*(−*bX*)) between *Pityophthorus juglandis* catches and light intensity (lux). (A) Female: *N* = 856, *F* = 14.90, *P*<0.01, *α* = 4.56, *b* = 0.0007, adj. *R* ^2^ = 0.48; (B) Male: *N* = 856, *F* = 8.31, *P*<0.05, *α* = 3.67, *b* = 0.0005, adj. *R* ^2^ = 0.33. Blue □: Outliers; Red ◯: Data points used to fit curves. These points were the greatest values of trap catches from each of the light intensity classes [28].

**Figure 6:**
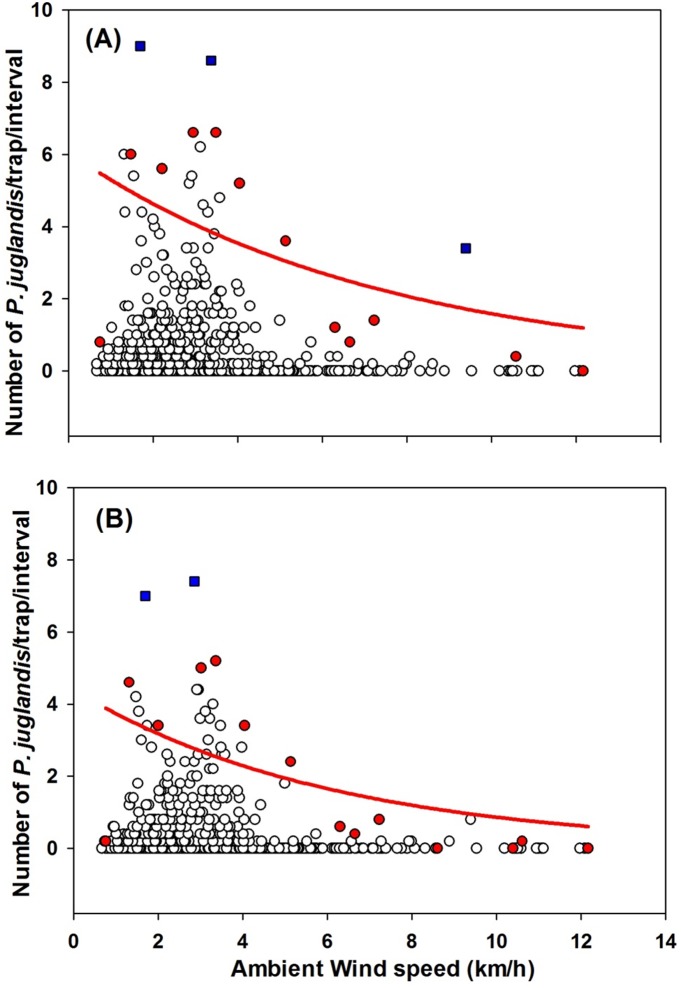
The exponential decay relationship (*Y* = *α*×*exp*(−*bX*)) between *Pityophthorus juglandis* catches and wind speed (km/h). (A) Female: *N* = 1205, *F* = 5.64, *P*<0.05, *α* = 6.06, *b* = 0.13, adj. *R* ^2^ = 0.30; (B) Male: *N* = 1205, *F* = 7.39, *P*<0.05, *α* = 4.40, *b* = 0.16, adj. *R* ^2^ = 0.33. Blue □: Outliers; Red ◯: Data points used to fit curves. These points were the greatest values of trap catches from each of the wind speed classes [28].

**Figure 7:**
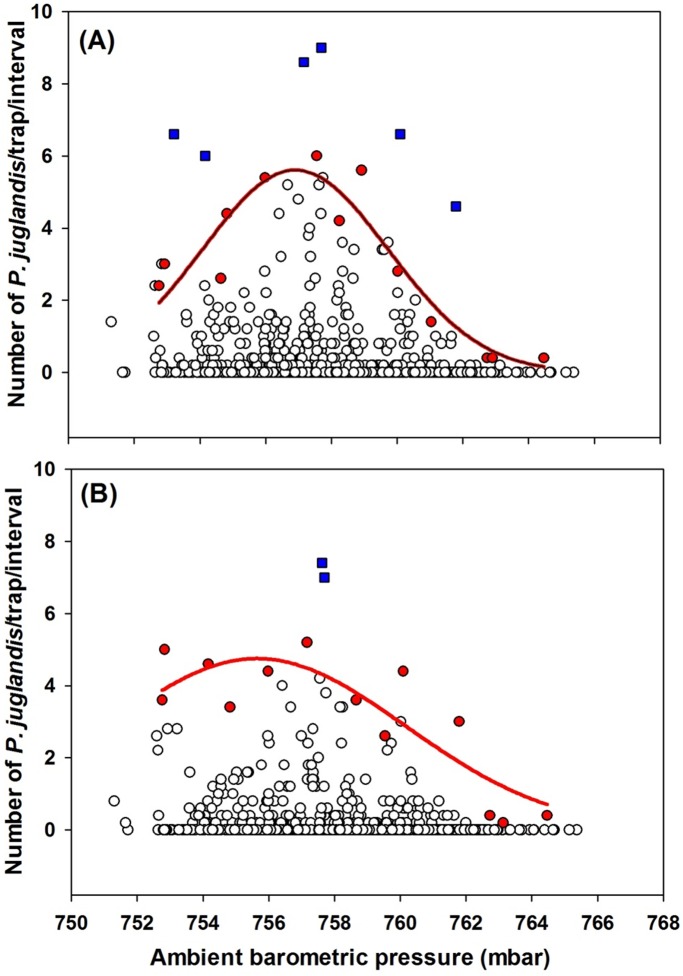
The Gaussian relationship (*Y* = *α*×*exp*(−(*X*−*X0*)^2^/2*b*)) between *Pityophthorus juglandis* catches and ambient barometric pressure (mbar). (A) Female: *N* = 873, *F* = 33.70, *P*<0.001, *α* = 5.60, *b* = 2.83, *X*_0_ = 756.90, adj. *R* ^2^ = 0.84; (B) Male: *N* = 856, *F* = 14.73, *P*<0.01, *α* = 4.75, *b* = 4.52, *X*_0_ = 755.65, adj. *R* ^2^ = 0.70. Blue □: Outliers; Red ◯: Data points used to fit curves. These points were the greatest values of trap catches from each of the barometric pressure classes [28].

From 8 May to 17 September, 2012, all abiotic variables varied significantly across day (Temperature: *X* ^2^ = 629.88; *df* = 8; *P*<0.001; Light: *X* ^2^ = 726.49; *df* = 8; *P*<0.001; Wind: *X* ^2^ = 266.57; *df* = 8; *P*<0.001; Barometric pressure: *F* = 14.36; *df* = 8, 845; *P*<0.001) (Fig. 3). Diurnal temperature ranged from 10 to 43°C (Fig. 4A, B). The temperature during the 1400 to 2000 h interval was the highest, followed by temperatures at 1200 and 2200 h (Fig. 3A). The lowest temperatures were recorded at 0600 and 0800 h (Fig. 3A). Light intensity ranged from 0 to 5,000 lux (Fig. 5A, B). Light intensity was greater between 1000 and 2000 h than at 0800 h, which was in turn more intense than at 0600 and 2200 h (Fig. 3B). Mean wind speed during the time intervals ranged between 0 and 12 km/h (Fig. 6A, B). The wind speed during the 1600 to 2000 h interval was greater than at any other time (Fig. 3C). Barometric pressure varied between 751 and 766 mbar (Fig. 7A, B). The pressures between 0800 and 1400 h were greater than at 0600, 1600, 1800, and 2200 h, which were in turn greater than at 2000 h (Fig. 3D).

**Figure 8:**
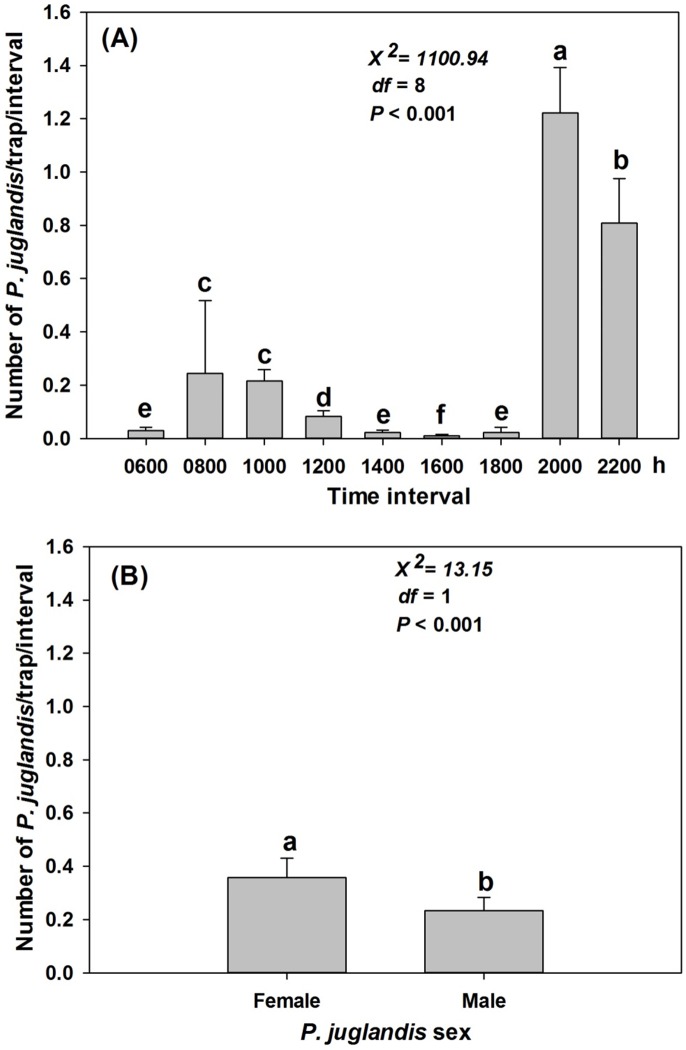
Effects of time interval of the day and *Pityophthorus juglandis* sex on *P. juglandis* catches (mean + SE). (A) Effect of time interval; (B) Effect of *P. juglandis* sex. Time intervals: 0600 h: 2200 h the previous day–0600 h the current day; 0800 h: 0600–0800 h; 1000 h: 0800–1000 h; 1200 h: 1000–1200 h; 1400 h: 1200–1400 h; 1600 h: 1400–1600 h; 1800: 1600–1800 h; 2000 h: 1800–2000 h; and 2200 h: 2000–2200 h. Different lower-case letters above bars denote significant difference between time intervals (A) or between *P. juglandis* sexes (B) at *α* = 0.05. *N*_time interval_ = 268 except the time interval 0800 when *N* = 266. *N*_sex_ = 1205 for both sexes. Means plotted in (B) represent catches per 2 h interval.

When the diurnal data were pooled between May and September, *P*. *juglandis* catches differed significantly with time of day (*X* ^2^ = 1100.94; *df* = 8; *P*<0.001). *Pityophthorus juglandis* flight showed a distinct bimodal pattern with the lower peak at 0800–1000 h and the higher peak at 2000–2200 h (Fig. 8A). During the higher peak, more *P. juglandis* were trapped at 2000 h than at 2200 h (Fig. 8A). The lowest *P. juglandis* catches were at 1600 h (Fig. 8A). Across all 2 h intervals, significantly more female than male *P. juglandis* were caught during the experiment (*X* ^2^ = 13.15; *df* = 1; *P*<0.001; Fig. 8B).

### Relationship between *P. juglandis* trap catch and individual abiotic variables

The Gaussian equation, *Y* = *α*×*exp*(−(*X*−*X0*)^2^/2*b*), fit the relationship between *P. juglandis* catches and temperature well, regardless of *P. juglandis* sex (Female: *F* = 63.38, *P*<0.001, α = 6.23, *b* = 5.11, *X*_0_ = 26.16, adj. *R* ^2^ = 0.89; male: *F* = 28.87, *P*<0.001, α = 4.37, *b* = 5.89, *X*_0_ = 26.87, adj. *R* ^2^ = 0.79; Fig. 4A, B). For populations of both sexes, peak *P. juglandis* flight occurred at around 26–27°C and flight was initiated at 11–12°C and terminated at 38–39°C (Fig. 4A, B). *Pityophthorus juglandis* catches were correlated with barometric pressure in a partial Gaussian manner, also irrespective of *P. juglandis* sex (Female: *F* = 33.70, *P*<0.001, α = 5.60, *b* = 2.83, *X*_0_ = 756.90, adj. *R* ^2^ = 0.84; male: *F* = 14.73, *P*<0.01, α = 4.75, *b* = 4.52, *X*_0_ = 755.65, adj. *R* ^2^ = 0.70; Fig. 7A, B). For both sexes, peak *P. juglandis* flight occurred at around 755–757 mbar (Fig. 7A, B).

*Pityophthorus juglandis* flight generally declined with increasing light intensity or wind speed. The exponential decay equation, *Y* = *α*×*exp*(−*bX*), fit the relationship between *P. juglandis* catch and light intensity well, regardless of *P. juglandis* sex (Female: *F* = 14.90, *P*<0.01, α = 4.56, *b* = 0.0007, adj. *R* ^2^ = 0.48; male: *F* = 8.31, *P*<0.05, α = 3.67, *b* = 0.0005, adj. *R* ^2^ = 0.33; Fig. 5A, B). When light intensity was considered alone, *P. juglandis* was most active below light intensities of 2000 lux. The equation, *Y* = *α*×*exp*(−*bX*), also fit the relationship between *P. juglandis* catch and wind speed well, regardless of *P. juglandis* sex (Female: *F* = 5.64, *P*<0.05, α = 6.06, *b* = 0.13, adj. *R* ^2^ = 0.30; male: *F* = 7.39, *P*<0.05, α = 4.40, *b* = 0.16, adj. *R* ^2^ = 0.33; Fig. 6A, B). When wind speed was considered alone, *P. juglandis* was most active between 1 and 4 km/h.

### Combined and interactive effects of abiotic variables on *P. juglandis* catches

**Figure 9:**
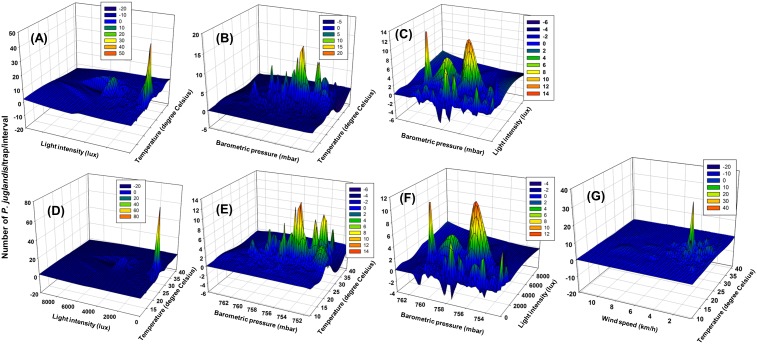
Second-order interactions of abiotic factors on *Pityophthorus juglandis* trap catches. (A) Temperature and light intensity (male); (B) Temperature and barometric pressure (male); (C) Light intensity and barometric pressure (male); (D) Temperature and light intensity (female); (E) Temperature and barometric pressure (female); (F) Light intensity and barometric pressure (female); and (G) Temperature and wind speed (female).

Model 25 had the minimum *QAIC* among the 94 models evaluated for female *P. juglandis* catches (Table 1). Model 44 also fit the data well (based on *w_i_*>0.10; *w_i_* indicates the relative likelihood of the model *i* being the best model given the data) for female catches. Both models included the first order of the four variables and three to four second-order interactions; parameters of almost all terms in both models were significantly different from zero (Table 2). Model 44 was the best and only model that fit well for *P. juglandis* male catches (Table 1). Parameters of almost all terms were significantly different from zero (Table 2). Relative importance (indicated by sum of *w_i_*) of terms in the model indicated that temperature, light intensity, wind speed, and barometric pressure were important determinants of *P. juglandis* catches, regardless of *P. juglandis* sex (Table 3). For female *P. juglandis*, among the six second-order interactions of the four variables, the interactions between 1) temperature and light intensity, 2) temperature and barometric air pressure, and 3) light intensity and barometric pressure were relatively the most important, followed by the interaction between temperature and wind speed (Fig. 9D–G). The interactions between light and wind speed, and between wind speed and pressure were relatively least important for females. Among the four third-order interactions for females, the interaction among light, wind speed, and pressure was relatively most important, and those among temperature, light, and pressure, and temperature, wind, and pressure were least important. For male *P. juglandis*, among the six second-order interactions, the interactions between 1) temperature and light intensity, and 2) temperature and pressure were relatively the most important. The interactions between 1) temperature and wind speed, 2) light intensity and wind speed, and 3) wind speed and pressure were relatively least important (Fig. 9A–C). Among the four third-order interactions, the interactions among temperature, light intensity, and wind speed, and among light intensity, wind speed, and pressure were relatively most important, whereas those among temperature, light intensity, and pressure, and among temperature, wind speed, and pressure were least important. For both sexes, the specific second-order interactions between the four abiotic factors were: flight peaked when temperature was between 25 and 30°C when light intensity was less than 2000 lux (almost no flight occurred at greater light intensity regardless of temperature) (Fig. 9A, D); flight peaked when temperature was between 25 and 35°C and barometric pressure was between 752 and 762 mba (and declined otherwise) (Fig. 9B, E); flight peaked when barometric pressure was between 755 and 761 mba and the light intensity was less than 2000 lux (and declined otherwise) (Fig. 9C, F); and flight peaked when temperature was ca. 30°C and wind speed was ca. 2 km/h (females only) (Fig. 9G).

**Table 1:** Summary of statistics from model selection to identify the best sets of models that predicted flight of *Pityophthorus juglandis* in response to ambient temperature (T), light intensity (L), wind speed (W), and barometric pressure (P).

Model number	*QAIC*	*Δ_i_*	*w_i_*	*ER*	Terms included in the model
Female					
Model 25	480.35	0	0.13	1.00	T, L, W, P
					T×L, T×W, T×P, L×P
Model 44	480.56	0.21	0.12	0.90	T, L, W, P
					T×L, T×P, L×P
Male					
Model 44	378.33	0	0.15	1.00	T, L, W, P
					T×L, T×P, L×P

Of all 94 formulated models (see Appendix S2), Models 25 and 44 were the best models (based on *w_i_*>0.10) that predicted female flight activity, whereas Model 44 was the best model that predicted male flight activity. *QAIC*: Quasi-likelihood adjusted Akaike’s Information Criteria (*AIC*); the model with the minimum *QAIC* was considered the best model; *Δ_i_* (* = QAIC_i_*–*QAIC_min_*): the plausibility that the fitted model is the best model given the data (the larger the *Δ_i_*, the less plausible is the model); *w_i_* indicates the relative likelihood of the model *i* being the best model given the data; *ER*: Evidence ratio, is the weight of the best model divided by the weight of the fitted model *j* (the closer the *ER* to 1 the better). Computation and interpretation of statistics followed [29]. See Appendix S3 (Female) and S4 (Male) for detailed statistics.

**Table 2:** Parameter (*β*s) estimation of the best set of models predicting flight of *Pityophthorus juglandis* in response to ambient temperature (T), light intensity (L), wind speed (W), and barometric pressure (P) and some of their interactions.

Parameter	Female				Male	
	Model 25		Model 44		Model 44	
	Estimate (SE)	*P*	Estimate (SE)	*P*	Estimate (SE)	*P*
T	−15.4431 (4.42)	<0.01	−16.7850 (4.36)	<0.01	−18.4993 (5.53)	<0.01
L	0.1542 (0.03)	<0.01	0.1534 (0.03)	<0.01	0.1226 (0.04)	<0.01
W	0.2789 (0.28)	>0.05	−0.2516 (0.07)	<0.01	−0.2387 (0.09)	<0.01
P	−0.3554 (0.15)	<0.05	−0.4057 (0.15)	<0.01	−0.5365 (0.19)	<0.01
T×L	−0.0001 (0.00)	<0.01	−0.0001 (0.00)	<0.01	−0.0001 (0.00)	<0.01
T×W	−0.0224 (0.01)	>0.05	–	–	–	–
T×P	0.0207 (0.01)	<0.01	0.0224 (0.01)	<0.01	0.0247 (0.01)	<0.01
L×P	−0.0002 (0.00)	<0.01	−0.0002 (0.00)	<0.01	−0.0002 (0.00)	<0.01

See Appendix S2 for detailed model components.

**Table 3:** Sum of *w_i_* (an indicator of relative importance in the model) for variables (terms) from selection of models predicting flight of *Pityophthorus juglandis* in response to ambient temperature (T), light intensity (L), wind speed (W), and barometric pressure (P) and their interactions.

Variable	Times included inmodels	Female	Male
First-order			
Temperature (T)	87	1.000^(1)^	1.000^(1)^
Light (L)	87	1.000^(1)^	1.000^(1)^
Wind (W)	87	1.000^(1)^	1.000^(1)^
Pressure (P)	87	1.000^(1)^	1.000^(1)^
Second-order interactions			
T×L	48	1.000^(1)^	1.000^(1)^
T×W	48	0.753^(2)^	0.575^(3)^
T×P	48	0.987^(1)^	0.957^(1)^
L×W	48	0.611^(3)^	0.560^(3)^
L×P	48	0.998^(1)^	0.875^(2)^
W×P	48	0.645^(3)^	0.561^(3)^
Third-order interactions			
T×L×W	9	0.266^(2)^	0.218^(1)^
T×L×P	9	0.191^(3)^	0.105^(2)^
T×W×P	9	0.166^(3)^	0.104^(2)^
L×W×P	9	0.361^(1)^	0.246^(1)^
Fourth-order interactions			
T×L×W×P	1	0.016^(1)^	0.008^(1)^

A total of 94 models (Appendix S2) were tested. *w_i_* indicates the relative likelihood of the model *i* being the best model given the data. Computation and interpretation of statistics followed [29]. Superscripted numbers in parentheses denote ranks of the sum of *w_i_* across terms appeared the same number of times in the models. See Appendix S3 (Female) and S4 (Male) for detailed statistics.

## Discussion

The seasonal portion of the study documented that *P. juglandis* generally initiated flight in late January and continued until late November in northern California. This broad pattern of seasonal flight could be divided approximately into three phases (emergence: January–March; primary flight: May–July; and secondary flight: September–October. *Pityophthorus juglandis* catches were female-biased (58.9%) with no apparent seasonally consistent shifts in the sex ratio of flying adults (Fig. 1B). A similar overall percentage of females was also recorded in the diurnal study between May and September 2011 (Fig. 8B). The female bias is not surprising because the lures in this study contained a single male-produced aggregation pheromone component. Temperature is generally the primary determinant of bark beetle emergence after completion of maturation feeding in host material [31], [32]. Intermittently occurring warm days during the northern California mid-winter may explain the initiation of annual emergence followed by flight in January by *P. juglandis*. Some trap catches recorded from January to March may have been low or zero because of days where high temperatures did not exceed ca. 17–18°C [23] or because only a small fraction of the bark beetle population may be captured in pheromone-baited traps [33]. The catches increased slowly over the following months as emergence rates likely increased and the primary flight began. The continuum of flight throughout the summer may be related to the re-emergence of parental adult beetles; late emergence of some fraction of the previous generation; and early development and emergence of some fraction of the current generation. Secondary flight (September−October) likely coincided with the emergence of the bulk of the current generation of *P. juglandis*. Catches were modulated throughout the summer in response to the life cycle and to the collective and interactive effects of ambient temperature, light intensity, wind speed, and barometric pressure (see below).

The diurnal portion of the study confirmed the bimodal pattern of flight behavior for *P. juglandis* [23]. This conclusion is supported by two measures of this flight tendency: 1) a significant number of days from the 134 day sample between May and September 2012 when *P. juglandis* was caught in flight in both the morning and afternoon/evening periods; and 2) by examination of the diurnal distribution of the pooled trap catches during the entire period of evaluation (Fig. 8A). Both unimodal [14]–[16], [34] and bimodal [11], [12], [17], [35] flight patterns of bark beetles have been documented. However, all previous studies that report diurnal modality have been short in duration. Mendel et al. [11] sampled for 3-d durations during four points in the flight season. During the winter season the diurnal pattern was unimodal, whereas during the other three periods, the diurnal pattern was bimodal. In our study, which was conducted during the spring and summer season, *P*. *juglandis* was captured in flight both in the morning and afternoon/evening hours in 87.1% (i.e., 108 of 124) of the days when at least one *P. juglandis* was caught. *Pityophthorus juglandis* was most active during the dusk period during the major part of the flight season (Figs. 2,8A). A minor peak in flight activity occurred during the middle morning hours. The flight pattern was consistent over the entire over the 4-mo study period. The switch of modality during late June/early July reported by Seybold et al. [23] was not observed in this study. The 13 days when no *P. juglandis* were caught in the morning, but at least one *P. juglandis* was caught in the afternoon/evening were scattered from May to August (May 10, 14, 15, 18, 20, 29; June 10, 22, and 26; July 17; and August 3, 5, and 7). The absence of the minor morning flight peak during late June/early July in Seybold et al. [23] might have been due to variability in environmental conditions (i.e., elevated morning temperatures) during that period in 2011 or the orchard habitat where the previous study took place. Also, in contrast to results reported in Seybold et al. [23], morning temperatures recorded at 0600 h in the present study did not significantly predict *P. juglandis* catches in the morning or the entire day.

Temperature [10], [23], [36], light intensity [10], [18], [36], wind speed [37]–[41], and barometric pressure [42], [43] have been reported individually or interactively to influence insect flight. When considering temperature in the absence of the other factors, Seybold et al. [23] reported that pheromone-guided flight of *P*. *juglandis* was modulated by ambient temperature in a Gaussian manner. Similarly, in the current study there were: (1) lower (ca. 11–12°C) and upper (ca. 38–39°C) flight thresholds and (2) a peak of flight activity at ca. 26–27°C (Fig. 4). The slight differences in temperature thresholds and peak temperature ranges between these two studies might be due to different lengths and seasonal intervals of the study periods or different regression methods used to analyze the data. Also, a portion of the data from Seybold et al. [23] was collected in an orchard habitat that was more open than the riparian forest studied here. In another well-studied bark beetle, the mountain pine beetle, *Dendroctonus ponderosae* Hopkins (Coleoptera: Scolytidae), lower and upper flight thresholds have been estimated as 19 and 41°C, respectively [19] with most beetles flying when temperatures are between 22 and 32°C [20], [35].

The interaction of insect flight and temperature may also be related to aspects of insect physiology. Many insects regulate body temperature [44]–[46]. The endothermic green darner dragonfly, *Anax junius* (Drury) (Odonata: Aeshnidae), likely regulates head and thoracic temperature during flight by decreasing wingbeat frequency and metabolic rate as temperature increases [46]. Likewise, the lower and upper flight thresholds of *P. juglandis* (and *D. ponderosae*) might be the cutoff points for cost-effective heat regulation. Alternatively, the thresholds might be the lower and upper limits of thermal regulation.

Almost all flight by *P. juglandis* occurred at light intensities below 2000 lux, which is ca. one twelfth to one fifth the light intensity in the midday of a typical overcast day (ranges between 10,000 and 25,000 lux; Wikipedia, http://en.wikipedia.org/wiki/Daylight, last accessed 7 June, 2014). Furthermore, as light intensity increased, *P. juglandis* flight activity decreased in the manner of the exponential decay equation, *Y* = *α*×*exp*(−*bX*) (Fig. 5). Low light intensity or darkness was associated with the cessation of *P. juglandis* flight activity; as the seasonal period of daylight shortened, *P. juglandis* flights between 2000 and 2200 h declined and ceased on 3 September, 2012 (Fig. 2C, green arrow). Darkness, however, was not the only determinant for cessation of *P. juglandis* flight since we had already recorded a persistent measure of zero light intensity at 2000 h beginning on 20 August, 2012, whereas *P. juglandis* flight activity during this interval continued until 3 September, 2013. One explanation for this disparity is that the quality (e.g., polarization) of the light affected the flight. Some Hymenoptera and Diptera utilize polarized light for orientation [47], but apparently the bark beetle, *D. ponderosae* does not [18]. Alternatively, initiation and cessation of flight by *P. juglandis* (and other insects) may be a consequence of the interaction of multiple environmental variables (discussed below).

As wind speed increased, *P. juglandis* flight also decreased in the manner of the exponential decay equation, *Y* = *α*×*exp*(−*bX*) (Fig. 6). Furthermore, most flight occurred at wind speeds between 1 and 4 km/h (i.e., 0.3–1.1 m/s). Catches of *Trypodendron lineatum* (Olivier) (Coleoptera: Scolytidae), a beetle more closely related to *P. juglandis*, dropped linearly as wind speed increased from 0.0 to 0.9 m/s (0.0 to 3.2 km/h) [40]. The maximum wind speed for flight of the Douglas-fir beetle, *Dendroctonus pseudotsugae* Hopkins, has been inferred to be approx. 2 m/s (7.2 km/h) [37]. Since many insects exploit airborne semiochemicals (primarily kairomones and pheromones) [48], [49], and volatiles in still air diffuse randomly, insects will most likely fly or walk randomly as *T. lineatum* does when there is no air motion [40]. Therefore, a modest range of wind speeds may assist insects with orientation during host- or mate-finding behaviors. High wind speeds, on the other hand, may interfere physically with insect flight or may reduce orientation behavior by transferring odor molecules too rapidly. In addition, the range of wind speeds for optimal flight may be dependent of the mass of an insect and its flight capacity.

The barometric pressure during our study of *P. juglandis* ranged between 751 and 766 mbar (Fig. 7), a range of less than 20 mbar. Regardless, *P. juglandis* responded overall to barometric pressure in a partial Gaussian manner: Flight increased as barometric pressure increased in the lower range and peaked at 755–756 mbar. The pattern was more evident for females than for males.

Studies on insect flight in response to abiotic environmental factors have focused almost exclusively on the individual factors or at most pairs of factors [18], [36], [50]–[52] and have rarely examined how combinations of multiple factors may act linearly and interactively to affect flight. Both male and female *D. ponderosae* were attracted to high light intensity (i.e., 60 watts) when temperature was below 35°C, but not to polarized light or when temperature was above 35°C [18]. Wind speed did not affect flights of *Capnodis tenebrionis* (L.) (Coleoptera: Buprestidae) at low temperatures, whereas it decreased flight at high temperatures [21]. The parasitoids *Trichogramma pretiosum* and *T. evanescens* (Hymenoptera: Trichogrammatidae) lowered their flight under rapid (i.e., 50 mbar in 1 h) barometric changes compared to slow or no changes (i.e., 50 mbar in 6 h), irrespective of the direction of change [43].

Model selection has been increasingly employed in the field of ecology and evolution [53], and two lines of evidence from the model selection procedure in the present study suggested the necessity for incorporating multiple factors and their interactions in quantitatively predicting *P. juglandis* flight. First, the best model(s) selected contained all four abiotic variables and either three or four second-order interactions (Table 1). The parameters (*βs*) of all terms included in the best model(s) were all significantly different from zero (except that of interaction between temperature and wind) (Table 2). Of the factors, temperature seemed to interact most frequently with other factors. Second, sums of the weights (*w_i_*) of the Akaike’s Information Criteria (AIC), an indicator of relative importance of the terms in the model, suggested that all four variables were of the same importance (Table 3). Of the second-order terms, those included in the best model(s) were of higher importance than the others. The sum of the AIC weights (*w_i_*) of some third-order interactions was greater than 0.2, which might also imply their importance.

The important second-order interactions (Fig. 9) in *P. juglandis* flight among the four abiotic factors were: 1) flight peaked when temperature was between 25 and 30°C when light intensity was less than 2000 lux and almost no flight occurred at greater light intensity regardless of temperature; 2) flight peaked when temperature was between 25 and 35°C and barometric pressure was between 752 and 762 mba (and declined otherwise); 3) flight peaked when barometric pressure was between 755 and 757 mba and the light intensity was less than 2000 lux (and declined otherwise); and 4) flight peaked when temperature was approx. 30°C and wind speed was approx. 2 km/h. Thus, an approach to understanding the mechanism for the largely crepuscular flight pattern of *P. juglandis* is to examine the composite (May−September) diurnal abiotic factors (Fig. 3) in comparison with the composite diurnal flight pattern (Fig. 8). The bimodal pattern of diurnal flight of *P. juglandis* (Fig. 8A) is best explained by diurnal patterns of moderate to relatively high temperature (Fig. 3A) that coincide with periods of low to intermediate light intensity (Fig. 3B) and periods of moderate to high wind speed (Fig. 3C). The highest periods of diurnal flight occurred when diurnal wind speed was high (Fig. 3C) and barometric pressure was relatively low (Fig. 3D).

For early detection as a prelude to a pest eradication or implementing an integrated pest management (IPM) program for *P. juglandis*, optimal trapping conditions are a combination of 1) ca. 26–27°C temperature; 2) less than 2000 lux light intensity; 3) ca. 755–757 mba barometric pressure; and 4) 1–4 km/h wind speed. Mating disruption or interruption of aggregation has become an important component of IPM programs for certain insect pests [54]–[57]. Efficacy of these methods is affected by semiochemical release rates [58], [59], which are in turn affected by abiotic factors such as temperature and wind speed. Understanding the interactions among abiotic environmental factors on flight activity could increase the efficacy of these methods in a specific IPM program for *P. juglandis*. Our new knowledge of the primary periods of seasonal flight by *P. juglandis* (May–July and September–October) provides some guidance for when semiochemical-based interruption of aggregation may be applied most efficaciously.

## Supporting Information

Appendix S1 **Raw data.**
(XLSX)

Appendix S2 **Model formulation.**
(XLSX)

Appendix S3 **Statistics for females.**
(XLSX)

Appendix S4 **Statistics for males.**
(XLSX)
